# Relationship of body weight with gastrointestinal motor and sensory function: studies in anorexia nervosa and obesity

**DOI:** 10.1186/s12876-016-0560-y

**Published:** 2017-01-05

**Authors:** Sena Bluemel, Dieter Menne, Gabriella Milos, Oliver Goetze, Michael Fried, Werner Schwizer, Mark Fox, Andreas Steingoetter

**Affiliations:** 1 Division of Gastroenterology and Hepatology, University Hospital Zurich, Raemistrasse 100, CH-8091 Zurich, Switzerland; 2 Institute for Biomedical Engineering, University and ETH Zurich, Zurich, Switzerland; 3 Menne Biomed Consulting, Tuebingen, Germany; 4 Psychiatric Department, University Hospital Zurich, Zurich, Switzerland; 5 Zurich Centre for Integrative Human Physiology (ZIHP), University of Zurich, Zurich, Switzerland

**Keywords:** Gastric emptying, Gastrointestinal transit, Visceral sensations, Anorexia Nervosa, Obesity

## Abstract

**Background:**

Whether gastrointestinal motor and sensory function is primary cause or secondary effect of abnormal body weight is uncertain. Moreover, studies relating continuous postprandial sensations of satiation to measurable pathology are scarce. This work assessed postprandial gastrointestinal function and concurrent sensations of satiation across a wide range of body weight and after weight change.

**Methods:**

Patients with anorexia nervosa (AN) and obesity (OB) were investigated in reference to normal weight controls (HC). AN were additionally investigated longitudinally. Gastric emptying, antral contractions and oro-cecal transit after ingestion of a solid meal were investigated by MRI and ^13^C-lactose-ureide breath test. The dependency of self-reported sensations of satiation on the varying degree of stomach filling during gastric emptying was compared between groups.

**Results:**

24 AN (BMI 14.4 (11.9–16.0) kg/m^2^), 16 OB (34.9 (29.6–41.5) kg/m^2^) and 20 HC (21.9 (18.9–24.9) kg/m^2^) were studied. Gastric half-emptying time (t_50_) was slower in AN than HC (*p* = 0.016) and OB (*p* = 0.007), and a negative association between t_50_ and BMI was observed between BMI 12 and 25 kg/m^2^ (*p* = 0.007). Antral contractions and oro-cecal transit were not different. For any given gastric content volume, self-reported postprandial fullness was greater in AN than in HC or OB (*p* < 0.001). After weight rehabilitation, t_50_ in AN tended to become shorter (*p* = 0.09) and postprandial fullness was less marked (*p* < 0.01).

**Conclusions:**

A relationship between body weight and gastric emptying as well as self-reported feelings of satiation is present. AN have slower gastric emptying and heightened visceral perception compared to HC and OB. Longitudinal follow-up after weight rehabilitation in AN suggests these abnormalities are not a primary feature, but secondary to other factors that determine abnormal body weight.

**Trial registration:**

Registered July 20, 2009 at ClinicalTrials.gov (NCT00946816).

**Electronic supplementary material:**

The online version of this article (doi:10.1186/s12876-016-0560-y) contains supplementary material, which is available to authorized users.

## Background

Clinically relevant over- and underweight states have been attributed to genetic, psychological and environmental factors that influence food intake [1–4]. The central perception of satiation and satiety regulates food intake and is modulated by biophysical and neurohormonal feedback mechanisms originating from the gastrointestinal (GI) tract [5–8]. By regulating digestive function, these signals link central perceptions with GI motility [9]. However, observational and interventional trials studying the interrelation between GI motor and postprandial sensory function with body weight have not provided definitive findings. Previous studies have focused on either under- or overweight patients. In addition, the interpretation of published data is difficult because the technologies applied to assess GI function (e.g., γ-scintigraphy, ^13^C-breath test) varied greatly between studies. Moreover, the relation of self-reported postprandial sensations of satiation and stomach volumes with body weight has not been demonstrated without invasive methods.

Individuals with morbid obesity have a delayed postprandial onset of satiation and less fullness [10]. Some authors suggest that this is related to relatively rapid gastric emptying compared to normal weight controls, but continuous assessments of sensations in the postprandial period are missing [11–15]. Moreover, observations varied between studies [16–21], so the role of altered gastric emptying and postprandial sensations in obesity remains controversial.

At the other extreme, many morbidly underweight patients with anorexia nervosa complain of prolonged fullness, bloating and nausea after meals [22]. These sensations have been linked to abnormal stomach function [23–26] – in particular, prolonged gastric emptying [25, 27–32]. However, the association between slow solid gastric emptying and clinically relevant postprandial symptoms is weak [33, 34] and it is not known whether this abnormality restricts meal consumption in this patient group.

This study aimed to clarify the relationship of postprandial GI motor and sensory function with body weight. The specific hypothesis tested was that the “GI response to feeding varies inversely with body weight” such that gastric emptying and oro-cecal transit increase and postprandial sensations of satiation decrease from under- to overweight. If present, then these findings would support the idea that the neurohormonal response to feeding is heightened in underweight and weakened in overweight patients and provide a physiological basis for the peripheral GI control of body weight (mediated by food intake). To this end, GI motor and sensory function in participants across a wide range of body weight including anorexic, normal weight and obese participants were investigated. In order to further test whether GI motor and sensory function are a primary cause or a secondary effect of abnormal body weight the participants with abnormal body weight were intended to be tested before and after weight rehabilitation.

## Methods

### Participants

Anorexia nervosa (AN) patients met DSM-IV criteria [35] and had a BMI <15.5 kg/m^2^ at inclusion. AN were recruited from an inpatient psychotherapy unit for patients with severe eating disorders of the Psychiatric Department of the University Hospital. After a somatic and psychiatric stabilization period at the Eating Disorders Centre of the Clinic for Psychiatry and Psychotherapy within the first 4 weeks following admission, the participants should regularly gain approximately 1 kg of weight per week. This was attained by interdisciplinary multimodal inpatients therapy, consisting of assisted meals, group and individual psychotherapy, body-perception therapy, creativity therapy, nutritional counselling and other therapies. Recruitment and visit 1 occurred within the first 2 to 4 weeks of the program (orientation phase), before patients start to gain weight. Visit 2 (group AN2) occurred after AN patients attained BMI >17.5 kg/m^2^. Normal weight, healthy controls (HC) with BMI 18.5–24.9 kg/m^2^ were recruited via public announcements. Obese participants (OB) with BMI >30 kg/m^2^ were recruited via the outpatient clinic of the Endocrinology Department of the University Hospital. Visit 2 was planned after patients lost >5% of body weight. Exclusion criteria were age <18 years and >60 years; history of gastrointestinal, cardiorespiratory (including hypertension), hematologic, renal or atopic disorders, diabetes, drug or alcohol abuse; abdominal surgery; regular intake of medication altering gut function (e.g. anticholinergics, laxatives, proton-pump inhibitors); presence of metallic foreign bodies interacting with magnetic resonance imaging (MRI); claustrophobia; body dimensions too large to fit into MRI scanner; pregnancy and lactation.

### Study design

**Fig. 1:**
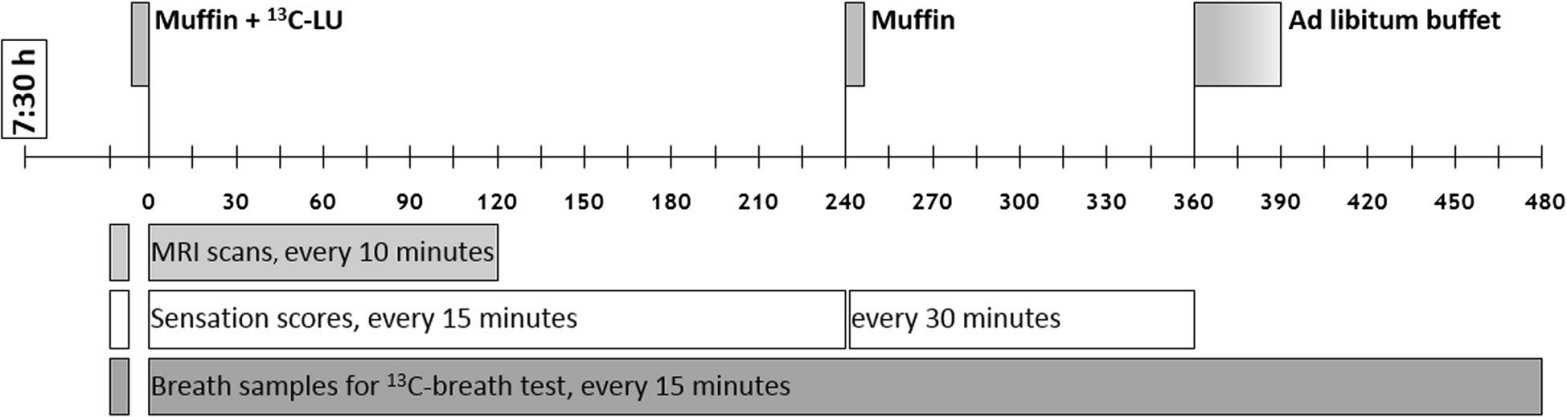
Overview of study day. Baseline measurements were acquired before ingestion of the ^13^C-labelled test meal. MRI scans of gastric content volume and motility, breath samples and sensation scores were acquired as indicated. ^13^C-LU = ^13^C-lactose-ureide breath test marker

At the screening visits for the cross-sectional and longitudinal comparison, participants meeting the inclusion criteria completed the short-form Leeds Dyspepsia Questionnaire (LDQ), Gastroparesis Cardinal Symptom Index (GCSI), Beck Depression Inventory II (BDI) and the State-Trait-Anxiety Inventory (STAI). Participants fasted for minimum eight hours and refrained from smoking prior to study days. On study days, women were tested for pregnancy by urine analysis, normogylcaemia was confirmed. Participants were not allowed to eat or drink apart from the offered food and beverages. As outlined in Fig. 1, following baseline measurements the muffin test meal (430 kcal, 21% fat, 63% carbohydrate, 16% protein) and the ^13^C-breath test marker were eaten together with 200 ml of tap water while seated on the MRI scanner table. After ingestion (time t = 0 min), participants were placed in supine position inside the scanner and postprandial gastric volumes, antral motility, breath samples and sensation scores were acquired at regular time intervals (ref. Fig. 1). After 240 min, participants had to eat another unlabelled muffin meal. After 360 min, an *ad libitum* buffet was provided consisting of water, tomato soup, cheese, apples, chocolate, crackers and butter.

### Magnetic resonance imaging

MRI was performed in a 1.5 T whole-body system (1.5 T Achieva; Philips Healthcare, The Netherlands). Gastric volume was derived from 30 axial MRI image planes covering the gastric region, performed during a single breath hold. Gastric content volume (GCV) was semi-automatically segmented at each time point using a custom designed image analysis software developed for MATLAB 7.4 (MathWorks Inc., Natick, MA, USA) [36]. Gastric motility scans consisted of 70 consecutive dynamic scans covering the gastric antrum and pylorus and recorded over 100 s during free-breathing [37].

### ^13^C-Lactose-ureide breath test

Oro-cecal transit time (OCTT) was assessed by ^13^C-lactose-ureide (^13^C-LU) (Euriso-Top, Saint-Aubin Cedex, France) breath test after priming of the colonic bacterial flora with five 100 milligram doses of unlabelled lactose-ureide the day prior to the study (validated by authors). On the study day 500 mg ^13^C-lactose-ureide blended into 2 grams of butter on 1x1 cm white bread was ingested immediately before the muffin meal [38]. Breath samples were collected into aluminized bags and analysed by non-dispersive isotope selective infrared spectroscopy (NDIRS, IRIS, Wagner Analysen Technik, Bremen, Germany). The increase of ^13^CO_2_ concentration in the exhaled air referenced to the baseline value (delta over baseline, DOB) was used for data analyses.

### Sensation scores

Participants were asked to rate their sensations of *hunger*, *fullness*, *nausea*, *bloating*, *abdominal pain*, *desire to eat* and *amount desired to eat* on a scale from 0 (*zero*, no sensation) to 10 (*ten*, maximal possible sensation) as described previously [39, 40].

### Data analyses and statistics

Group size and power calculations were based on the primary study outcome measurement: half-time of gastric emptying (t_50_). Published MRI data reported a mean difference of 29 min in t_50_ between a solid and a liquid test meal in 8 healthy participants, which was used as a clinically relevant effect size [41]. To detect the same difference with α = 0.05 and power 90%, the required group size was estimated to be 16. Data plots and statistical analyses were performed with the software R, version 2.13.1 (R Foundation for Statistical Computing, Vienna, Austria).

MRI gastric content emptying curves were fitted by a power-exponential function to derive t_50_ [42]. To compensate for heteroscedasticity (reduction of the number of outliers and homogenization of variances), values of t_50_ were log-transformed for linear model analysis. Differences in log(t_50_) between groups are given as ratios (with confidence intervals). Gastric motility data were analyzed by a linear mixed-effect model.

OCTT was determined from the DOB-versus-time plots by a web-based consensus application (mean from four separate raters). Results were censored at OCTT >480 min. Analysis was based on Kaplan-Meier estimates and log-rank (Mantel-Haenszel) tests.

LDQ and GCSI were analyzed as previously described [43, 44]. The LDQ asks for the frequency and severity of indigestion, heartburn, regurgitation and nausea within the preceding 2 months. Each item scores 0 to 4, and the sum (0 to 32) indicates severity of dyspeptic symptoms. The GCSI scores 0 to 5 for nausea, retching, vomiting, stomach and excessive fullness, inability to finish a meal, loss of appetite, bloating, and abdominal distention. A mean score of <3 indicates mild, a score of 3.0 to 3.9 moderate and a score of ≥4.0 severe gastroparesis symptoms [45]. BDI and STAI were analyzed according to the German manuals [46, 47]. The BDI evaluates self-reported depression symptoms using 21 questions. Each answer scores between 0 and 3, and the sum of scores indicates depressive symptoms (≤12: no depression symptoms, 13–19: mild symptoms, 20–29 moderate symptoms, ≥30 severe symptoms). The STAI uses 20 questions to evaluate “state” anxiety (temporary emotion) and 20 questions to evaluate “trait” anxiety (anxious personality). Both parts are interpreted separately, and each answer scores between 1 and 4. The higher the score, the higher the anxiety. Differences in distributions of questionnaire scores and demographic data between the groups were analyzed by Kruskal-Wallis test. Healthy controls were expected to have the lowest scores.

Differences in initial group-wise sensation scores were tested with a bootstrapped median test because of the highly skewed distribution (package *pairwiseCI* for program R) [48]. To analyze the dependency of sensations of satiation on gastric content volume, the cumulative link mixed model package *ordinal* and function *clmm2* for the program R were applied. Linear fits were computed to the cumulative logits (ordinal logistic regression) of *hunger* and *fullness* scores with group as fixed effect [49]. This was followed by a logistic transformation in order to obtain the cumulative probability that a participant chooses a defined satiation score.

All tests based on linear models were corrected for 3-fold multiple testing using the Tukey method. Computed data are given as mean and 95% confidence interval (CI) unless otherwise stated.

## Results

### Demographics and descriptive statistics

The study was conducted between October 2010 and July 2012. Demographic data of the study population included in the cross-sectional comparison are given in Table 1. The majority of patients in all groups were female. The age in the obese participant group was wider spread (*p* < 0.001). The distributions of dyspepsia, anxiety and depression scores (ref. Table 1) were different between groups (all *p* < 0.01).

**Table 1:** Demographic and descriptive data of study population for the cross-sectional comparison

	AN	HC	OB
# participants (men)	24 (0)	20 (3)	16 (5)
Age [years]	23 (18–41)	24 (18–38)	32 (19–56)^a^
BMI [kg/m^2^]^b^	14.4 (11.9–16.0)	21.9 (18.9–24.9)	34.9 (29.6–41.5)
LDQ^b^	7 ± 2.6	1.3 ± 1.1	4.1 ± 2.0
GCSI^b^	2.1 ± 1.4	0.4 ± 0.6	0.7 ± 0.8
BDI^b^	26.0 ± 9.6	3.5 ± 2.0	7.6 ± 7.5
STAI (state)^b^	62.6 ± 8.6	45.1 ± 7.6	54.2 ± 6.2
STAI (trait)^b^	66.4 ± 10.1	45.0 ± 7.2	51.9 ± 10.6

Demographic data are given as mean (range); descriptive data are given as mean ± standard deviation

^a^ indicates a significant different distribution of values in the labelled group compared to unlabelled groups

^b^ indicates a significant different distribution between all groups (Kruskal-Wallis test, all *p* < 0.01)

12 of 24 AN patients included in the cross-sectional study were re-investigated after 112 (69 to 161) days of weight rehabilitation. These AN2 patients achieved a mean BMI increase of 3.4 (2.7 to 4.1) kg/m^2^ to a mean BMI of 18.1 kg/m^2^. The individual changes between visits for dyspepsia, anxiety and depression are displayed in Additional file 1: Figure S1. No participant with OB lost sufficient weight to meet the criteria for re-investigation during the study period.

### Gastrointestinal motor function

**Fig. 2:**
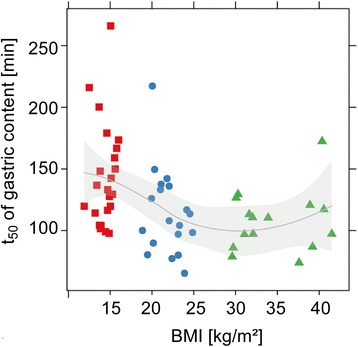
Gastric emptying (t_50_) over the full range of body weight (BMI). Participant groups are indicated by squares (participants with anorexia nervosa), dots (healthy controls) and triangles (participants with obesity). The solid line and shaded band show a smooth fit and the 95%-confidence range

GI motor function was determined by MRI (assessment of gastric emptying and antral contraction frequency) and ^13^C-lactose-ureide breath test (oro-cecal transit). Respective t_50_ values were: AN 138.7 (121.3 to 158.5) minutes, HC 110 (94.9 to 127.4) minutes and OB 105.5 (89.4 to 124.5) minutes. The ratio of log-transformed t_50_ data showed slower gastric emptying in AN patients compared to HC and OB participants (HC:AN = 0.8 (0.7 to 1), *p* = 0.016; OB:AN = 0.8 (0.6 to 0.9), *p* = 0.007 and OB:HC = 1.0 (0.8 to 1.2), *p* = 0.89). A correlation between body weight and gastric emptying was observed up to a BMI of 25 kg/m^2^ (*p* = 0.0068), but not at higher values (Fig. 2). All groups had similar mean antral contraction frequencies (~3/min; *p* > 0.3).

Oro-cecal transit time showed a tendency to decrease with increasing body weight, but median values were statistically not different: AN = 346 (294 to NA) minutes, HC = 308 (277 to 430) minutes and OB = 280 (266 to 401) minutes (log-rank test over all groups, *p* = 0.564).

In the longitudinal study, after weight rehabilitation in AN, gastric emptying time slightly decreased. The average t_50_ was 134.3 (109.9 to 148.4) and 121.5 (99.5 to 134.3) minutes for AN1 and AN2, respectively. The ratio of log-transformed values of t_50_ was 0.90 (0.82 to 1.00), *p* = 0.087. Mean antral contraction frequency did not change. For AN1 it was 2.7 (2.5 to 3)/min and for AN2 2.8 (2.6 to 3)/min, *p* = 0.45.

### Gastrointestinal sensory function

**Fig. 3:**
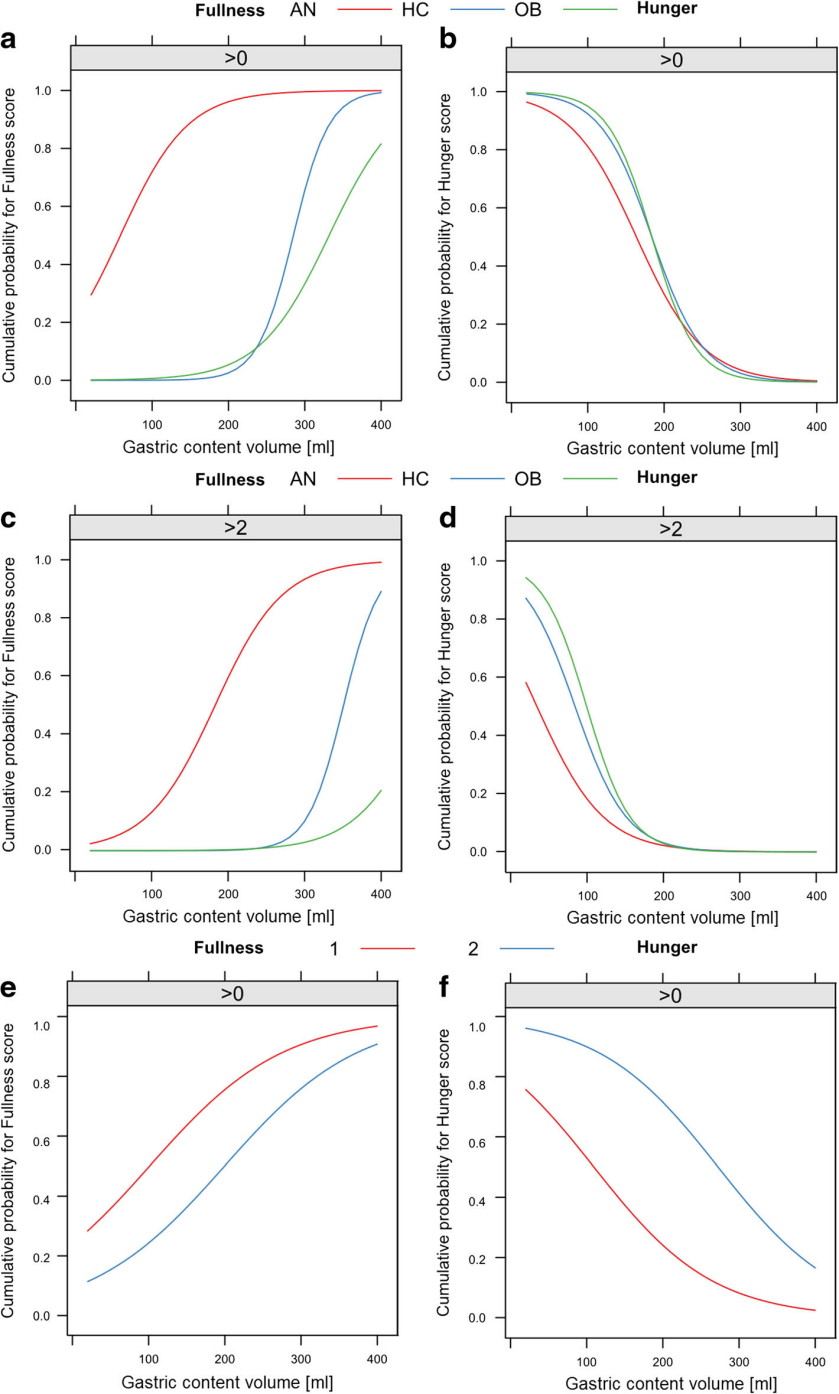
Sensation scores for *hunger* and *fullness* in relation to gastric content volume. The plots display the probability (y-axis) that participants report *fullness* or *hunger* higher than a stated threshold (indicated above each panel) at a given gastric content volume (x-axis). The maximal postprandial content volume after the meal is plotted at the right of the x-axis. Compared to HC, the sensation of *fullness* reported by AN patients was significantly shifted to the left (i.e., increased visceral sensation) and for OB the relationship was shifted to the right (i.e., decreased visceral sensation). This is illustrated for *fullness* scores *> zero* (**a**) and > *two* (**c**). Conversely, at any given volume, the sensation of *hunger* reported by HC and OB was significantly greater than that reported by AN patients. This is illustrated for *hunger* scores *> zero* (**b**) and > *two* (**d**). The 12 AN patients included in the longitudinal analysis sensed less *fullness* (**e**) and more *hunger* (**f**) after the weight rehabilitation program. 1 = AN at visit 1, 2 = AN at visit 2

GI sensory function was determined by self-reported sensation scores and food intake at an *ad libitum* buffet. Postprandial sensations of *nausea*, *bloating* and *abdominal pain* were rarely reported after the test meal and were not different between groups. The dependency of self-reported sensations of *fullness* and *hunger* on the varying postprandial gastric content volume is displayed in Fig. 3. *Fullness* was maximal immediately after food intake (Fig. 3a and c). Postprandial *fullness* was greater in AN patients than in HC and OB participants at any given volume (*p* < 0.001). For example, all AN patients still rated *fullness* > *zero* when gastric content volume was 200 ml, whereas median *fullness* in HC dropped to *zero* between gastric content volume 300 to 250 ml. In contrast, OB participants occasionally rated *fullness* as *zero* even at maximal gastric volume after the meal.

The sensation of *hunger* was reported by all groups when postprandial gastric content volume decreased below 300 to 250 ml (ref. Fig. 3b and d). In AN patients, *hunger* increased more slowly as gastric emptying proceeded than in HC and OB participants (*p* < 0.04).

After weight gain, sensation scores in AN2 for both *fullness* and *hunger* were shifted towards larger gastric content volumes (*p* < 0.001), i.e. less abnormal values (ref. Fig. 3e and f).

Satiety was measured by *ad libitum* food intake, with higher satiety corresponding to lower food intake. AN ate less than HC and OB; however, there was no difference between OB and HC (HC-AN: 376 (178 to 574) kcal, *p* < 0.001; OB-AN: 511 (292 to 731) kcal, *p* < 0.001; OB-HC: 135 (-90 to 361) kcal, *p* = 0.33). AN preferred to eat carbohydrates, while HC and OB ate comparable amounts of fat and carbohydrates (detailed data on file). After weight rehabilitation, AN2 patients increased food intake by 6.1% (*p* = 0.034). The composition of food ingested remained unchanged.

## Discussion

Postprandial gastrointestinal (GI) motor and sensory function was investigated across a wide spectrum of body weight. A relationship was detected between body weight and gastric emptying such that anorexic patients (AN) had slower gastric emptying than healthy controls (HC) or obese participants (OB). The dependency of postprandial sensations of satiation on the changing gastric content volume during emptying was extracted and displayed using an ordinal logistic regression approach based on the concurrent and continuous MRI volume data and sensation ratings. For any postprandial gastric content volume, AN reported markedly more *fullness* and less *hunger* compared to HC and OB participants. Taken together, these findings confirmed the hypothesis that GI response to feeding varies inversely with body weight. The additional longitudinal follow-up in anorexic patients demonstrated that both gastric emptying and sensations can become less abnormal following weight rehabilitation.

### Gastrointestinal motor function and body weight

A relationship between gastric emptying and body weight was present in the BMI range from 12 to 25 kg/m^2^, corresponding to AN patients and HCs. These findings are consistent with previous studies that often reported delayed gastric emptying for AN, but similar emptying in OB compared to HC [16–19, 25, 27–31, 50–52]. Interestingly, no difference in antral contraction frequency was present between groups. In this regard, it can be inferred that relatively slow gastric emptying in AN patients is not due to impaired breakdown (grinding) of the solid test meal by antral contraction waves, but due to other factors such as enhanced nutrient feedback from the small intestine. The oro-cecal transit time (OCTT) documented by ^13^C-lactose-ureide breath test followed the same pattern as gastric emptying, i.e., it decreased with increasing BMI from AN to HC and OB. However, no statistical difference between groups was detected, due to high variability of OCTT. The latter might result from an undiagnosed small intestinal bacterial overgrowth (not tested during screening) that would result in a premature rise of ^13^CO_2_ in the exhaled air. Previous studies have also reported normal or prolonged OCTT in AN [53, 54] with no consistent abnormality in OB patients [16, 55, 56]. The rate of gastric emptying and OCTT is thought to be modulated by secretion of GI peptide hormones (e.g. CCK, GLP-1, PYY) in response to nutrient sensing in the small bowel (termed “the ileal brake”) [16, 29, 57–59]. The relevance of the major GI peptide hormones for gastric emptying and satiation in the diseased state, i.e., AN and OB compared to HC, will be evaluated by state-of-the-art multivariate modelling. Since this adds sizeable complexity to the data analyses, it was considered beyond the focus of this work. Besides any direct effects of GI peptide hormones, the differences in GI motor function for AN may also be explained by different dietary habits at study entry. As AN patients notoriously avoid fatty food (also observed during *ad libitum* buffet), it can be assumed that the muffin represents a high-fat meal for this group [60, 61]. Acute fat consumption following a fat-restricted diet has been shown to prolong gastric emptying in lean and obese humans [62–64]. Conversely, with chronic high-fat diet the gastric emptying rate increases over time [65, 66]. These effects are thought to be due to up- and down-regulation of intraluminal fat digestion and mucosal fat sensing, respectively, impacting on the neurohormonal GI response (“ileal brake”) [66–68]. However, here the relationship of GI motility and nutritional habits remains speculative, as this study did not control for long-term food intake. The influence of female sex steroid hormones on gastric emptying might add to the observed differences in gastric emptying between the groups. While some studies could not find differences between the follicular and luteal phase of the menstrual cycle in women, other studies showed slower gastric emptying in premenopausal women investigated in the follicular phase and postmenopausal women taking hormone replacement [69–71]. This was accompanied by reduced contractility in the gastric antrum [71]. Here, the study days were not timed with the menstrual cycle of the female participants. However, a difference in antral contractions was not observed between groups. Furthermore, AN participants were amenorrheal and the majority took oral contraceptives. If this has the same effect on gastric emptying as hormone replacement in postmenopausal women has not yet been investigated.

In the longitudinal investigation of anorexic patients, a trend to faster gastric emptying after successful weight rehabilitation was observed. It should be noted that AN2 patients were still underweight (average BMI of 18.1 kg/m^2^) and that this effect might have been more distinct with more pronounced increase in BMI. This finding is consistent with previous studies in AN with comparable follow-up time and BMI at follow-up [25, 27, 29, 50, 72]. These investigations found accelerated gastric emptying particularly in the restrictive type of anorexia nervosa, while gastric emptying in the purging type remained unchanged (here 9 of 12 patients that gained weight were classified as restrictive type AN at study entry). While AN patients at visit 1 had a restrictive eating behaviour, all AN2 patients were close to discharge and ate regularly a balanced, ca. 2400 kcal diet. Thus, at re-investigation the test meal was more similar to the habitual diet than at study entry, thereby contributing to a more normal gastric emptying. Psychological comorbidities, like anxiety or major depression, can additionally influence gastrointestinal motility [73]. This was addressed in a covariable analysis with data from 8 AN patients with complete data sets for t_50_, BDI and STAI for both study visits (Additional file 1: Figure S1). None of the tests with this small data set showed evidence for an effect of STAI and BDI on the estimated differences in t_50_. Recruitment of constitutionally thin and overweight individuals with comparable dietary habits and without psychiatric comorbidities could possibly overcome such confounders [74].

Whereas 50% of the AN patients attained the pre-defined weight change required for re-investigation, this was not achieved by any of the OB patients. This could have several reasons. First, the out-patient consultations of OB did not include behavioural and psychotherapy. As OB are less persistent and self-directive than AN patients [75, 76], this could have contributed to the failure of the weight rehabilitation program. Second, during the study period bariatric surgery became a health insurance paid treatment for BMI ≥35 kg/m^2^, which might have reduced the motivation in some OB participants to lose weight conventionally [77].

Taken together, the results of the longitudinal study suggest that abnormal gastric emptying is not a primary feature of underweight, but more likely secondary to other factors (e.g. eating habits, diet, psychiatric comorbidities). However, the lack of control for these factors does not allow for discrimination between these possibilities.

### Gastrointestinal sensory function and body weight

Important differences in self-reported postprandial sensations of satiation were present between the three study groups. By combining continuous MRI volume data with postprandial sensation ratings and using an ordinal logistic regression approach, *fullness* and *hunger* ratings could directly be related to the degree of gastric filling after intake of the ~400 ml muffin test meal (Fig. 3a-d). Previous MRI studies described a linear relationship between fullness and gastric distention in healthy participants and patients with functional dyspepsia [78, 79]. The presented approach allows for a more detailed observation of this relationship without using invasive techniques such as barostat. This allowed the following conclusions: (i) Most HC sensed an “empty” stomach (i.e., *fullness* ratings ~ *zero*) while the stomach still contained 200 ml and started to report *hunger* (i.e., *hunger* ratings > *zero*) at a gastric content volume of <100 ml. (ii). Interestingly, a number of OB patients did not feel any *fullness* even at maximal stomach filling; however, overall, the sensation of *fullness* and *hunger* was comparable to HCs. (iii) In contrast, AN patients report heightened sensitivity to gastric filling compared to HC and OB patients with approximately 1 in 3 AN patients still reporting *fullness* and no *hunger* at all when the stomach was completely empty. Consistent with this experimental data, questionnaires also demonstrated more dyspeptic and gastroparesis symptoms in AN patients (Table 1). Additionally, satiety, as assessed by the *ad libitum* buffet, was also more marked in AN patients than in HC and, in particular, OB patients. These findings complement a previous study in obese individuals that reported reduced *fullness* at one single postprandial time point compared to normal weight controls [10]. In AN, a previous epidemiological study found severe postprandial dyspeptic symptoms, i.e., fullness and early satiety [22].

The relationship between sensations of satiation and gastric distention is mediated by gastric mechanosensitive receptors with the former being modulated by the macronutrient composition (especially fat content) of the ingested meal [79–81]. Chemo-sensitive nutrient sensors in the small intestine trigger the release of GI peptides (e.g. CCK, GLP-1, PYY). These act as neuro-hormones that modulate vagal afferent activity and central perception [5, 82, 83]. For example, CCK enhances the satiating effect of gastric filling (i.e., reduces hunger) and high postprandial CCK levels may explain the altered sensation of *fullness* and *hunger* in AN patients [83–85]. However, a meta-analysis of studies in AN patients revealed only slightly increased CCK levels at baseline compared to HC, but equivalent levels after meals [86]. Thus, analogous to the effects on gastric emptying, altered perception of *fullness* in AN and OB might not be related to direct effects of GI peptide hormones on gastric function, but rather to the habitual diet at study entry. It has been shown that chronic, high-fat diet leads to a decrease in postprandial *fullness* and *satiety* respectively [80, 87]. Therefore, it may be the composition of the test meal relative to the habitual diet of participants that explains the observed differences. Psychiatric comorbidity provides several other, possible explanations for differences in *fullness* and *hunger* sensations between the study groups. First, anxiety among AN patients (Table 1) could reduce gastric accommodation which would be expected to increase postprandial *fullness* [88]. Second, reduced reporting of *hunger* and higher *satiety* ratings may represent a “secondary gain” in AN patients undergoing weight rehabilitation [27]. Third, interoceptive awareness and visceral sensitivity are decreased in AN patients, resulting in diminished ability to discriminate *hunger* and *satiety* sensations [89, 90]. In summary, abnormal reports of sensations in this group could be exaggerated by abnormal GI physiology, attempts by patients to excuse eating less, or the psychiatric disease and related comorbidities.

Longitudinal investigation of AN1 vs. AN2 revealed that *fullness* and *hunger* ratings shifted towards more normal levels (Fig. 3e and f). There was also less *satiety*, as indicated by increased intake at the *ad libitum* buffet. As discussed above, such improvements could be the result of physiological adaptation to increased oral intake or behavioural change. Previous studies found an improvement of interoceptive awareness in AN patients with weight gain [91]. However, it is not possible to assess from study results whether this is a consequence of weight rehabilitation, adaption to regular food intake or improved psychological state. Of note, food intake increased by only 6%, thus, the trend to more normal postprandial sensations is more likely related to regular food intake and weight gain, than to psychological rehabilitation. The latter is also mirrored by unchanged anxiety, i.e., values of the STAI (state). Indeed, if the latter occurred, then one would expect AN patients to choose chocolate or cheese rather than apples and soup at the *ad libitum* buffet, which was never observed.

## Conclusions

By investigating participants across a wide range of BMI, this study provides detailed insight into the relationship of GI motor and sensory function with body weight. Results indicate that gastric emptying rate is decreased and postprandial sensations of satiation are increased in AN patients compared to HC and OB patients. In principle, these findings support the hypothesis that the physiological response to feeding is heightened in lower body weight. The improvement in gastric emptying and postprandial sensations of satiation in AN after weight rehabilitation suggest that the differences in GI motor and sensory function are unlikely to be a primary cause of abnormal body weight. Secondary causes, such as dietary habits and psychiatric comorbidities, are more likely to determine GI motor and sensory function in the investigated groups.

## Additional file

Additional file 1: Figure S1. Longitudinal comparison of AN patients. Displayed are the data of the 12 participants that were investigated at visit 1 and visit 2. Paired data: *n* = 11 for LDQ and GCSI; *n* = 9 for BDI and STAI. (TIF 10832 kb)

## Abbreviations

AN: Patients with anorexia nervosa
AN2: AN at follow-up (after weight rehabilitation)
BDI: Beck Depression Inventory II
BMI: Body mass index
GCSI: Gastroparesis Cardinal Symptom Index
GI: Gastrointestinal
HC: Healthy controls
LDQ: Leeds Dyspepsia Questionnaire
MRI: Magnetic resonance imaging
OB: Patients with obesity
OCTT: Oro-cecal transit time
STAI: State-Trait-Anxiety Inventory
t_50_: Gastric content half-emptying time
