# Supplementary figures and images for: Relationship of body weight with gastrointestinal motor and sensory function: studies in anorexia nervosa and obesity
Source: BMC Gastroenterol. 2017 Jan 5;17:4. doi: 10.1186/s12876-016-0560-y (PMC5217542; doi:10.1186/s12876-016-0560-y)

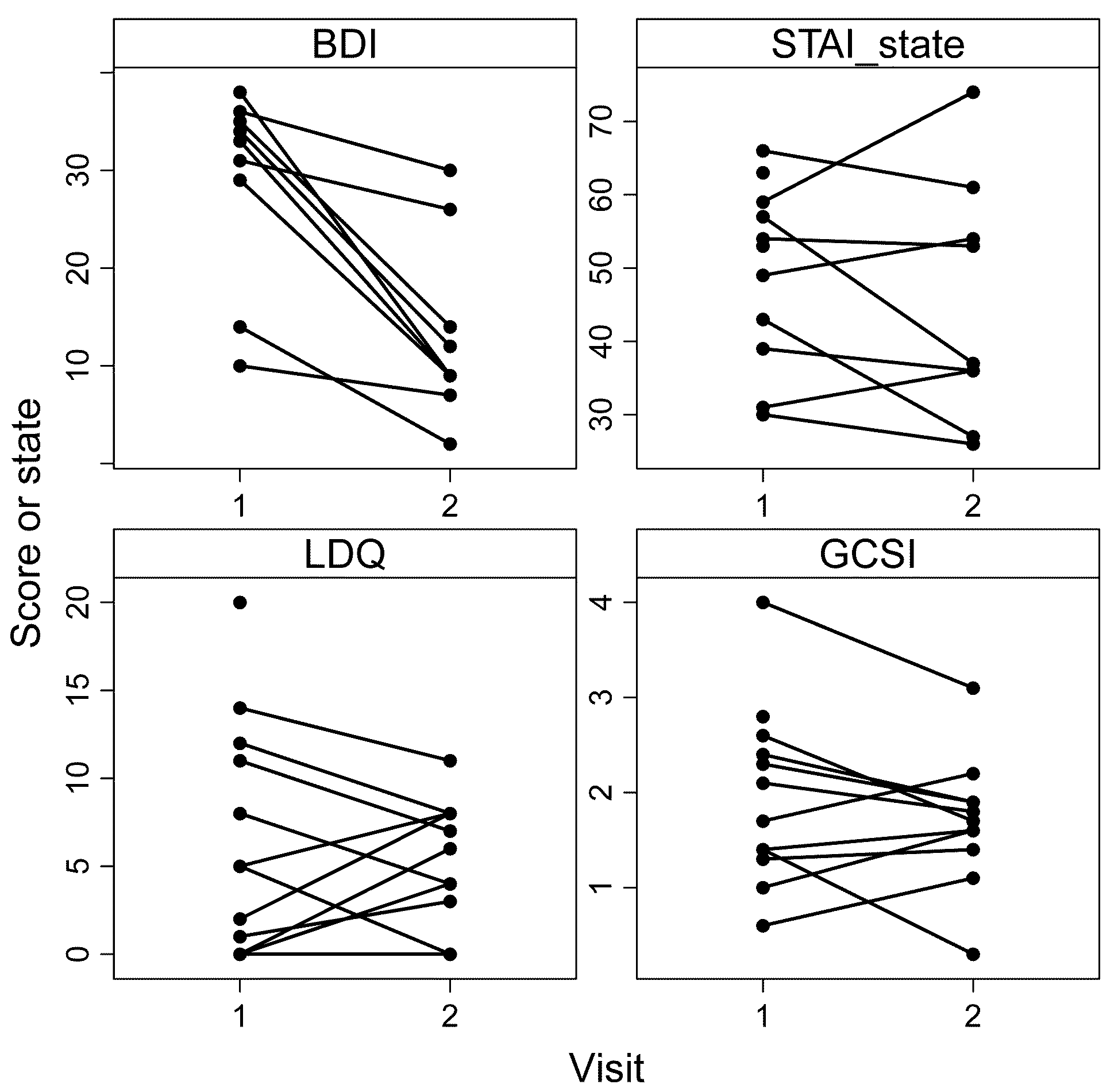

Supplement: Additional file 1: Figure S1. — Longitudinal comparison of AN patients. Displayed are the data of the 12 participants that were investigated at visit 1 and visit 2. Paired data: n = 11 for LDQ and GCSI; n = 9 for BDI and STAI. (TIF 10832 kb) [file 12876_2016_560_MOESM1_ESM.tif]
